# A Numerical Model for Simulating the Hemodynamic Effects of Enhanced External Counterpulsation on Coronary Arteries

**DOI:** 10.3389/fphys.2021.656224

**Published:** 2021-04-12

**Authors:** Bao Li, Ke Xu, Jincheng Liu, Boyan Mao, Na Li, Hao Sun, Zhe Zhang, Xi Zhao, Haisheng Yang, Liyuan Zhang, Tianming Du, Jianhang Du, Youjun Liu

**Affiliations:** ^1^Department of Biomedical Engineering, Beijing University of Technology, Beijing, China; ^2^The School of Life Sciences, Beijing University of Chinese Medicine, Beijing, China; ^3^Department of Cardiac Surgery, Peking University Third Hospital, Beijing, China; ^4^Philips (China) Investment Company, Shanghai, China; ^5^The Eighth Affiliated Hospital, Sun Yat-sen University, Shenzhen, China

**Keywords:** enhanced external counterpulsation, coronary artery, 0D/3D geometric multi-scale model, vascular endothelial cells, hemodynamic effects

## Abstract

Traditional enhanced external counterpulsation (EECP) used for the clinical treatment of patients with coronary heart disease only assesses diastolic/systolic blood pressure (Q = D/S > 1.2). However, improvement of the hemodynamic environment surrounding vascular endothelial cells of coronary arteries after long-term application of EECP is the basis of the treatment. Currently, the quantitative hemodynamic mechanism is not well understood. In this study, a standard 0D/3D geometric multi-scale model of the coronary artery was established to simulate the hemodynamic effects of different counterpulsation modes on the vascular endothelium. In this model, the neural regulation caused by counterpulsation was thoroughly considered. Two clinical trials were carried out to verify the numerical calculation model. The results demonstrated that the increase in counterpulsation pressure amplitude and pressurization duration increased coronary blood perfusion and wall shear stress (WSS) and reduced the oscillatory shear index (OSI) of the vascular wall. However, the impact of pressurization duration was the predominant factor. The results of the standard model and the two real individual models indicated that a long pressurization duration would cause more hemodynamic risk areas by resulting in excessive WSS, which could not be reflected by the change in the Q value. Therefore, long-term pressurization during each cardiac cycle therapy is not recommended for patients with coronary heart disease and clinical treatment should not just pay attention to the change in the Q value. Additional physiological indicators can be used to evaluate the effects of counterpulsation treatment.

## Introduction

Enhanced external counterpulsation (EECP) is a non-invasive method that uses air-inflated cuffs to mechanically compress the human lower body and increase the diastolic blood pressure (DBP). This decreases compression at the onset of systole and decreases the vascular resistance to reduce the intra-aortic systolic blood pressure (SBP) (Applebaum et al., 1997). By improving blood circulation, EECP assists cardiac function and increases blood perfusion in the heart and brain as well as the kidneys and other organs. By inflating the air cuffs wrapped around the lower body and buttocks during diastole, EECP can promote the backflow of blood from the limb arteries to the aorta, significantly increasing the DBP. Simultaneously, cardiac output (CO) is improved due to increased blood flow return to the heart. At the end of diastole, the air cuffs are quickly deflated. The pressure is released, reducing the distal afterload of the heart and accelerating the blood flow to the global circulation, thereby achieving a counterpulsation effect.

As a non-invasive medical device, the hemodynamic effect of EECP is significant in the treatment of ischemic vascular diseases. Numerous clinical studies (Michaels et al., 2002; Sharma et al., 2013; Qin et al., 2016) have shown that the acute hemodynamic effects of EECP can effectively improve the overall functional state of the blood circulatory system using “double pulse” perfusion, which effectively causes revascularization and improves the symptoms associated with organ ischemia. The markedly increased aortic pressure (AP) and CO during counterpulsation also help increase coronary blood flow. At present, for patients with coronary heart disease, the clinical treatment goal of EECP is that the ratio of diastolic blood pressure to systolic blood pressure is greater than 1.2 (Q = D/S > 1.2). When Q is greater than 1.2, it indicates that the DBP has increased to a level that is sufficient to meet the blood perfusion demand associated with myocardial ischemia. However, the evaluation of treatment effects based on this indicator lacks the scientific basis of mechanobiology theory because EECP accelerates blood flow, which improves the local hemodynamic environment of coronary artery vascular endothelial cells (VECs).

Additional researches (Wu et al., 2006; Zhang et al., 2007) have revealed that the improvement in WSS was the underlying mechanism in treating atherosclerosis, which was the long-term hemodynamic effect of EECP on VECs. WSS is related to the activation of endothelial Ca^2+^ and eNOS (Ilkan et al., 2017). The application of EECP will increase Ca^2+^, plasma NO levels, and promote eNOS gene expression of VECs by increasing WSS (Zhang et al., 2007). Therefore, the long-term hemodynamic effects can be reflected by the variation in WSS during EECP.

Previous studies (Malek et al., 1999; Zhang et al., 2007; Samady et al., 2011; Eshtehardi et al., 2012; Speelman et al., 2016) have shown that WSS is an important indicator that significantly impacts vascular remodeling and inhibits the development of atherosclerosis. The formation and development of atherosclerosis result from the long-term deposition of extracellular lipids in the intima of blood vessel walls (Libby, 2001). The increase in WSS (1–7 Pa) will lead to a hemodynamic environment that is conducive to alter the gene expression of VECs, thus reducing lipid deposition and vascular inflammation. After long-term treatment, the vascular endothelium will be smooth and exhibits good patency, which promotes benign remodeling of diseased blood vessels. Additional research (Pipp et al., 2004) has shown that high WSS can promote the growth of collateral vessels that had stopped growing, which significantly increases the numbers of new microcirculatory vessels around the stenotic region. Therefore, a high WSS around the stenosis might increase blood perfusion in the ischemic region through blood flow separation. Both processes described above are the long-term hemodynamic effects of EECP therapy.

Vascular endothelia, whose pathological conditions are closely related to the surrounding hemodynamic environment, are the target of EECP. Long-term EECP treatment continually exposes VECs to a mechanical environment that includes high WSS and a low oscillatory shear index (OSI). High WSS (1-7 Pa) will significantly affect the gene expression of VECs, affecting cell growth and metabolism, modifying the cellular immune inflammatory response, inhibiting VEC apoptosis and weakening intracellular lipid deposition, thereby protecting the vascular intima and inhibiting the development of atherosclerosis. In contrast, low WSS (<1 Pa) promotes atherosclerosis (Malek et al., 1999; Samady et al., 2011; Eshtehardi et al., 2012). Excessive WSS (>7 Pa) will damage VECs and cause positive remodeling and outward expansion of blood vessels. For patients with unstable plaques, excessive WSS can even cause plaque rupture, resulting in the formation of a thrombus and even acute myocardial infarction (Fu et al., 2015).

OSI is a mechanical index that reflects unstable blood flow. A high OSI (>0.2) indicates more backflow, which is a risky hemodynamic environment for the vascular endothelium and a sign of potential development of atherosclerosis and vulnerable plaques (Bassiouny et al., 1992; Mori et al., 2002; Ladisa et al., 2010; Speelman et al., 2016). However, EECP treatment can reduce the probability of backflow in coronary arteries. Therefore, an improved hemodynamic environment for VECs following long-term EECP therapy can effectively promote benign vascular remodeling and improve vascular function, which are the long-term hemodynamic effects of EECP treatment. Therefore, it is essential to research the quantitative long-term hemodynamic effects of EECP.

Clinical EECP therapy consists of a range of different counterpulsation modes. Currently, it is not clear how different counterpulsation modes alter the hemodynamic effects on coronary arteries. There are no published clinical studies that describe how the different counterpulsation modes intervene in the local hemodynamic environment of coronary VECs. Using a numerical simulation, Du formulated an ideal three-dimensional (3D) model to simulate the WSS variations of local atherosclerotic plaques under the intervention of EECP, which proved the effect of counterpulsation on the WSS of the vascular wall (Du and Wang, 2015). Xu conducted a numerical simulation of EECP based on real coronary arteries that exhibited different degrees of stenosis (Xu et al., 2020). They observed that during the counterpulsation state, the hemodynamic risk area in the stenotic coronary artery was decreased significantly, which proved that EECP produced a significant inhibitory effect on coronary atherosclerosis. However, the existing models have not been used to simulate the different counterpulsation modes. Also, the various neural regulation mechanisms of the human body during the counterpulsation state have not been considered.

In this study, by coupling the lumped parameter model (0D model) of the blood circulatory system and the 3D model of a coronary artery, a 0D/3D geometric multi-scale model was established. During counterpulsation, the model considered the body’s baroreflex mechanism, the autoregulation mechanism of coronary blood flow, and the vascular collapse of the lower body. We presented a more comprehensive method for the numerical simulation of EECP, and the model was verified using clinical experiments. By applying different counterpulsation modes in the 0D model, this study explored the long-term hemodynamic effects of EECP on the coronary vascular endothelium, and provided a mechanobiology-based theory for the clinical long-term therapeutic effects.

## Materials and Methods

### Geometric Multi-Scale Model of the Coronary Artery

Geometric multi-scale modeling is a specialized strategy used to simulate the blood circulatory system. The strategy uses different types of models to simulate different parts of the circulatory system. The 3D model can be used to provide detailed observations of the local hemodynamic environment of the blood vessel. The peripheral circulatory system can be simulated using a 1D model that considers the one-dimensional geometric information of the blood vessel or a 0D lumped parameter model that completely reduces the dimensionality of the segmented vessel. The 0D model can be used to provide non-artificially determined missing values as boundary conditions for the calculation of the 3D model, such that when the 3D model structure changes, the boundary conditions provided by the peripheral 0D model also will be adaptively changed to avoid the adverse effects by fixed boundary conditions. With the 3D model as the main focus, a geometric multi-scale model can simulate large-scale or even the entire circulatory system based on less computational cost (Kim et al., 2010; Taylor et al., 2013).

Using 3D modeling tools, such as Mimics (a CTA image segmentation software) and Freeform (a tactile interaction system), 3D models of coronary arteries were constructed. The algorithm for 0D/3D modeling and calculation in this study was based on the fluid mechanics simulation software ANSYS. The finite element simulation of the 3D model was based on the CFX module. The simulation of the 0D model was based on the secondary development function, User Junction Box Routine, provided by ANSYS-CFX. The simulation of the 0D model was programmed into this function by FORTRAN. Using the 0D/3D coupling algorithm (Zhao et al., 2016), the geometric multi-scale model can be solved after setting the initial conditions and time step. The 0D model simulation solution used the explicit Euler method, an explicit integral method with simple programming form and clear meaning, with a time step of 0.00001 s. The 3D model simulation used the finite element method based on the Navier-Stokes equation. The flow was assumed to be laminar and the time step was 0.001 s. This was the maximum time step needed to maintain the convergence, accuracy and efficiency of the computation. The choice of time step was based on the different time step experiments, which were introduced in our previous study (Li et al., 2019b, Biomed. Eng. Online). In the calculation process for the 0D model, linear interpolation was performed on the missing values caused by the mismatched time step to realize the data interaction between the two models. The interaction utilized the following formula:

(1) $${\bar{P}}_{3\,D,i\,n}=\frac{1}{A_{3\,D,i\,n}}\,\int_{\tau_{i\,n}}P\,d\tau =P_{0\,D,i\,n}$$

(2) $$Q_{3\,D,o\,u\,t}=\rho \,\int_{\tau_{o\,u\,t}}\mu \,n_{i}\,d\tau =Q_{0\,D,o\,u\,t}$$

where ${\bar{P}}_{3\,D,i\,n}$ is the mean inlet pressure calculated by the 3D model, *A*_*3D,in*_ is the inlet area of the 3D model, τ_*in*_ is the integral domain (the inlet plane of the 3D model), *P* is the pressure of each element on the inlet plane of the 3D model, *d*τ is the differential area element, and *P*_*0D,in*_ is the missing value of the 0D model, which is the mean inlet pressure of the 3D model. *Q*_*3D,out*_ is the outlet flow calculated by the 3D model, ρ is blood density, τ_*out*_ is the integral domain (the outlet plane of the 3D model), μ is the node velocity of the outlet plane of the 3D model, *n_i* is the normal vector of the outlet plane and *Q*_*0D,out*_ is the missing value of the 0D model (the outlet flow of the 3D model). In the coupling algorithm, data exchange between the two models was conducted during each time step of the 3D calculation, and residual detection was performed at the same time. The error of the outlet pressure and the error of the inlet flow rate of the 3D model between adjacent cardiac cycles were defined as the residual detection items. When the residuals were less than the pre-set value, the calculation result was considered to be convergent and the simulation ended. In addition, during the calculation process of the 0D model, the missing items in the boundary provided by the 3D model calculation must be interpolated to ensure the synchronization of the calculation time step of the two models. Because the time interval between each step was very short, linear interpolation was adopted.

**FIGURE 1:**
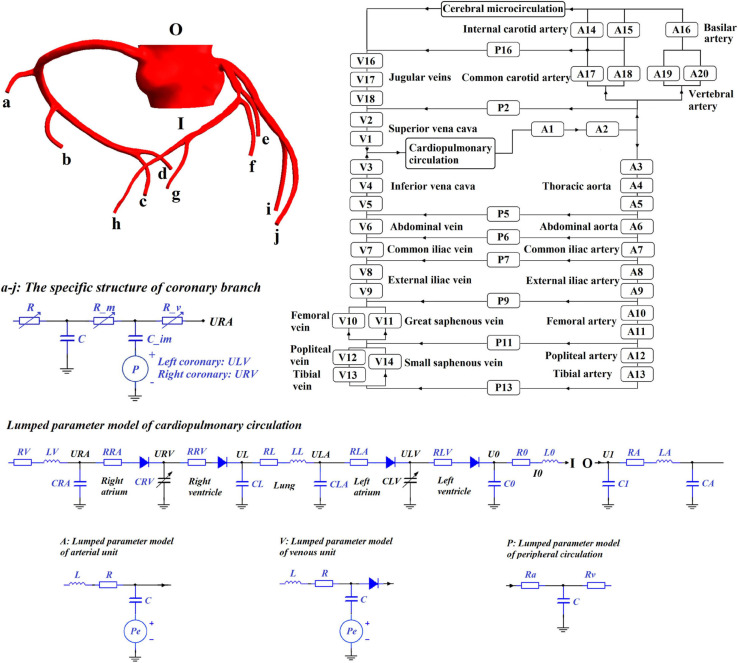
Universal 0D/3D geometric multi-scale standard model of a coronary artery.

This study chose the 0D/3D multi-scale model to simulate the hemodynamic responses of the cardiovascular system during the application of EECP, as shown in Figure 1. Since the purpose of this study was to quantitatively explore the effects of different counterpulsation modes on long-term cardiovascular hemodynamics, the methods and research conclusions, methodologically speaking, should be applicable to most patients but not designed for specific individuals. Therefore, the established cardiovascular geometric multi-scale model, should be able to represent the majority of people, rather than confined to a particular location or specific lesion. In this paper, this model was named the standard model. Therefore, a disease-free coronary artery 3D model was reconstructed for hemodynamic analysis to study the impact of different counterpulsation modes. Also, referring to previous studies (Jaron et al., 1988; Bai et al., 1992, 1994), a closed-loop 0D lumped parameter model of the human blood circulatory system was established. The model consisted of 20 arterial units, 17 venous units, eight peripheral circulation units, one cerebral microcirculation unit and one cardiopulmonary circulation unit. Among these units, the voltage source Pe, which existed in the arterial and venous units, was used to simulate the counterpulsation pressure, which only existed in the calves, thighs and buttocks (A8–A13 and V8–V14). The values for vascular compliance C, flow resistances R and flow inertia L in each unit of systemic circulation of the standard model were shown in our previous study (Li et al., 2019b, Biomed. Eng. Online). The values of C in the coronary circulation also were shown in the previous study (Li et al., 2019a, Med. Biol. Eng. Comput.). The initial values were calculated using the classical formulas of lumped parameter modeling as follows (Wang et al., 1989; Pietrabissa et al., 1996):

(3) $$C=\frac{3\,\pi \,r^{3}\,l}{2\,E\,h},R=\frac{8\,\pi \,\mu \,l}{A^{2}},l=\frac{\rho \,l}{A}$$

where *r* is the vessel radius, *l* is the length of the vessel segment, *E* is the Young’s modulus of the vessel, *h* is the wall thickness of the vessel, *μ* is the blood viscosity, *A* is the cross-sectional area and *ρ* is the blood density. The material properties of each vessel segment were described in a previous study (Liang et al., 2009). When applied to the standard model, the initial values of these parameters needed to be adjusted using an optimization algorithm (Li et al., 2020), which is the same as the parameter acquisition methods of individual models. The optimization objectives of the standard model were physiological indicators observed in most people (aortic pressure was 80–120 mmHg and cardiac output was 5 L/min). The characteristics of the geometric multi-scale model indicated that it was suitable for the hemodynamic simulation of EECP because when counterpulsation was applied in the 0D model, the local details in the 3D model were observed in real-time to evaluate variations in the local hemodynamic environment.

### Numerical Simulation of EECP

**FIGURE 2:**
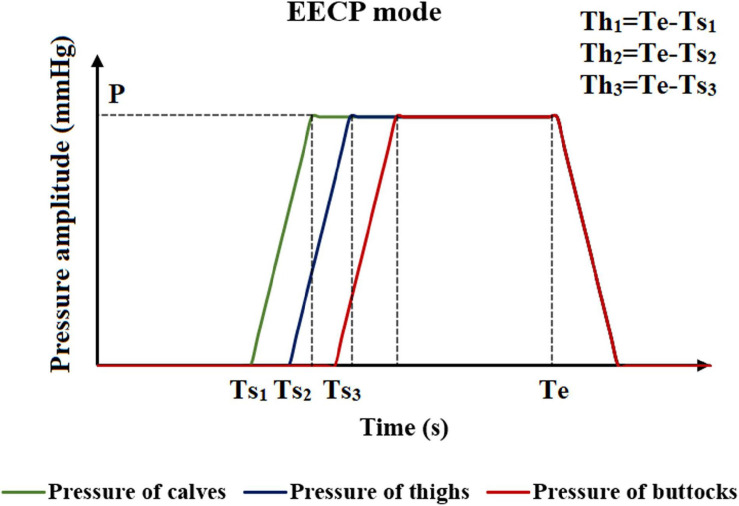
Schematic diagram of the EECP mode. Where Ts_1_, Ts_2_, and Ts_3_ are the moments when the counterpulsation cuffs of each part start to inflate. P is the pressure amplitude, and Th_1_, Th_2_, and Th_3_ are the pressurization durations of counterpulsation cuffs. Te is the deflation moment.

The method of applying pressure is based on four different parameters, including inflation and deflation rate, starting moment of pressurization, pressurization duration, and pressure amplitude. The control chart is shown in Figure 2. The inflation and deflation of the cuffs were set at a rapid rate in the clinical EECP instrument. In addition, considering the previous study (Bai et al., 1994), the inflation and deflation times in this study were both 0.005 s. The starting moment of the cuffs’ pressurization in clinical operation was triggered based on the R wave of the ECG, which was the starting point of systole during a cardiac cycle. After a delay of approximately 0.25 s, that is, the end of the systolic period, the cuffs were sequentially inflated. Therefore, the starting point of pressurization of the calves in this study was set at 0.25 s of the cardiac cycle. Based on clinical experience, the interval between the starting points of each part was 0.05 s (Bottom, 1999). Therefore, the starting moments of pressurization of calves, thighs and buttocks were 0.25, 0.30, and 0.35 s, respectively. Unlike the first two parameters, the pressurization duration and pressure amplitude significantly affected the treatment effect of EECP and should be carefully considered. Different counterpulsation modes also depended on these two parameters. In this study, the long-term hemodynamic effects of EECP on coronary artery VECs were studied by simulating different pressure amplitudes and pressurization durations. The pressure amplitudes of the three pressurization parts (calves, thighs, and buttocks) were synchronized (Li et al., 2019b, Biomed. Eng. Online.).

When using a lumped parameter model to complete the calculation, the pressure solving equations in calves, thighs and buttocks (units A8–A13 and V8–V14) during the resting state were as follows:

(4) $$d\,u=\frac{i}{C}\,\text{⋅}\,d\,t$$

where *du* is the differentiation of pressure to time, *i* is flow, *C* is capacitance, and *dt* is the time step. During EECP, the capacitances in A8-A13 and V8-V14 were connected to an external pressure source, and the solving equation becomes:

(5) $$d\,u=\frac{i}{C}\,\text{⋅}\,d\,t+d\,P_{e}$$

where *dPe* is the differentiation of the counterpulsation pressure to time.

### Neural Regulation Mechanisms During EECP

During EECP, the body’s aortic DBP is greatly increased, and the baroreflex is activated to maintain the balance of blood pressure and blood flow. The baroreflex is an adaptive regulation mechanism based on sympathetic nerve and vagus nerve signal stimulation. By dilating blood vessels, as well as regulating systemic peripheral vascular resistance, heartbeat frequency, cardiac contractility and systemic venous volume, the baroreflex exhibits dynamic behavior when the blood pressure changes. With reference to previous researches (Ursino, 1998; Liang and Liu, 2006), the baroreflex during EECP was included in our 0D model. The numerical simulation of the baroreflex was based on the sympathetic nerve and vagus nerve signals. The sympathetic signal is a monotonic exponential function negatively correlated with afferent neural pathway activity, which can be mathematically described as follows:

(6) $$f_{e\,s}=f_{e\,s,\infty }+\left(f_{e\,s,0}-f_{e\,s,\infty }\right)\,\text{⋅}\,e^{-k_{e\,s}\,\text{⋅}\,f_{c\,s}}$$

where *f*_*es*_ is the frequency of spikes in the efferent sympathetic nerves, *k*_*es*_, *f_*es*_,_0_*, and *f_*es*_,_∞_* are constants and *f*_*cs*_ is the frequency of spikes in the afferent fibers. The vagal signal is a positive function correlated with the activity in the sinus nerve, which can be mathematically described as follows:

(7) $$f_{e\,v}=\frac{\left[f_{e\,v,0}+f_{e\,v,\infty }\,\text{⋅}\,\exp\left(\frac{f_{c\,s}-f_{c\,s,0}}{k_{e\,v}}\right)\right]}{\left[1+\exp\left(\frac{f_{c\,s}-f_{c\,s,0}}{k_{e\,v}}\right)\right]}$$

where *f*_*ev*_ is the frequency of spikes in the efferent vagal fibers, and *k*_*ev*_, *f_*ev*_,_0_*, *f_*ev*_,_∞_* and *f_*cs*_,_0_* are constants. The value of all parameters and the mathematical description of *f*_*cs*_ were presented in the reference (Ursino, 1998).

Based on the stimulation of the two signals, the heart rate, systemic peripheral vascular resistance, cardiac contractility and systemic venous capacity were adjusted during the process of EECP simulation according to the following equations:

(8) $$\sigma_{\theta }\,\left(t\right)\,\left\{ \begin{array}{c} G_{\theta }\,\text{⋅}\,l\,n\,\left[f_{e\,s}\,\left(t-D_{\theta }\right)-f_{e\,s,m\,i\,n}+1\right]\,\text{if}\,f_{e\,s}\geq f_{e\,s,m\,i\,n}\\ 0\;\text{if}\,f_{e\,s}<f_{e\,s,m\,i\,n} \end{array}\right.$$

(9) $$\frac{d\,△\,\theta }{d\,t}\,\left(t\right)=\frac{1}{\tau_{\theta }}\,\text{⋅}\,\left(-△\,\theta \,\left(t\right)+\sigma_{\theta }\,\left(t\right)\right)$$

(10) $$\theta \,\left(t\right)=△\,\theta \,\left(t\right)+\theta_{0}$$

where θ is the generic-controlled parameter, *σ_θ_* is the output of the static characteristic, *τ_θ_* is the time constant and *D*_θ_ is pure latency. *f*_*es,min*_ is the minimum value of the sympathetic signal and *G*_θ_ is a constant gain index. All parameters were specified in the reference (Ursino, 1998). In addition to the above method, there are other contemporary modeling methods of baroreflex regulation that can be adopted. (Olufsen et al., 2006; Matzuka et al., 2015).

**FIGURE 3:**
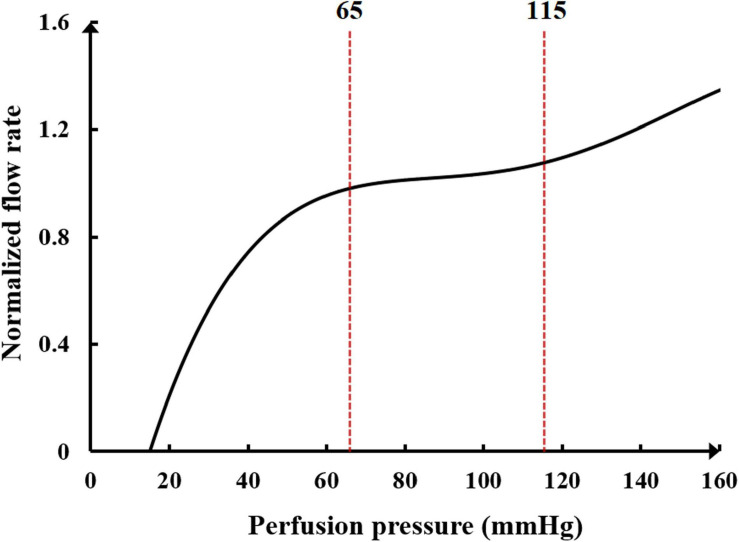
The relationship between normalized blood flow and perfusion pressure in the coronary flow autoregulation.

Another neural regulation influence on the cardiovascular system during the application of counterpulsation is the autoregulation of coronary blood flow. This mechanism is an adaptive regulation function of coronary blood vessels to maintain the nearly constant blood flow through myogenic, shear-dependent, and metabolic vascular responses to changes in perfusion pressure (Waters et al., 2011). Using animal experiments, Mosher observed that a sudden change in perfusion pressure caused a corresponding change in coronary blood flow, but the blood flow would automatically recover to the original level in approximately 30 s to 2 min due to the rapid adjustment response of coronary capillaries (Mosher et al., 1964). However, when the perfusion pressure was excessive, the autoregulation function was reduced considerably, resulting in a non-linear increase in the flow rate. The trend of normalized blood flow and perfusion pressure is presented in Figure 3.

It can be seen in Figure 3 that the perfusion pressure range for the coronary artery to maintain stable blood flow is between 65 and 115 mmHg. When the average perfusion pressure is greater than 120 mmHg, it is difficult for the coronary artery to maintain a stable blood flow. A mathematical model to simulate the coronary autoregulation (Ge et al., 2018) is:

$$R^{k+1}=R^{k}[K_{p}cor\left(k\right)+K_{i}\sum_{j=0}^{k}$$

(11) $$cor\left(j\right)+K_{d}\left(cor\left(k\right)-cor\left(k-1\right)\right)]$$

where

(12) $$c\,o\,r\,\left(k\right)=1+\frac{Q^{k}-Q^{R}}{Q^{R}}$$

In the formula, *R*^*k*^ and *R^*k*+1^* represent the small arterial resistance of the *k*th and next iteration steps, respectively. *cor(k)* is the correction function of *R*^*k*^, which is calculated as the difference between the simulated average coronary flow (*Q*^*k*^) and the target value (*Q*^*R*^, average coronary flow corresponding to the coronary perfusion pressure in Figure 3). *K*_*p*_, *K*_*i*_, and *K*_*d*_ are gain coefficients, which were set to 0.5, 0.01, and 0.01, respectively. These parameters were described in previous research (Ge et al., 2018). However, several parameters were modified to be suitable for the model in this study. This mathematical model was applied to the coronary microcirculation resistance in Figure 1. The resistances were changed in real-time at each iteration step to simulate the coronary autoregulation during the EECP state.

This model also considered the effect of vascular collapse when EECP was applied to the lower body. In a standard three-parameter windkessel model of a blood vessel segment, when the external pressure on the vessel was greater than the threshold value of the vessel collapse, the capacitance of the vessel will break down and the pressure of counterpulsation will act on the circuit directly just as a stabilized voltage supply because of the compliance loss after vessel collapse. The critical value was different for each person. This modeling method was discussed in our previous study (Li et al., 2019b, Biomed. Eng. Online., Li et al., 2020).

### Verification by Clinical Experiments

The 0D/3D geometric multi-scale model of the coronary artery and the numerical simulation model of EECP established in this study is a standard model suitable for most people. However, for each patient, the model needed to be individualized to explore the hemodynamic laws of EECP that were applicable for that individual. To verify the accuracy and effectiveness of the numerical calculation model and determine whether it was applicable to each individual, two individuals volunteered to carry out the clinical trials. To avoid uncertain risk during EECP, we selected two young (20–30 years old) healthy individuals without coronary heart disease, atherosclerosis, heart disease, hemorrhagic disorders or any vasculitis. The trials were approved by the ethics committee of the Peking University Third Hospital. In the experiments, we collected the coronary CTA images of the subjects to reconstruct the 3D model of the coronary arteries. Then, to ensure that the simulated results of the model were suitable for each individual, the AP waveforms and CO data of the subjects were collected during the resting state to individualize the 0D model of the blood circulatory system using an optimization algorithm (Zhang et al., 2016; Li et al., 2020). By coupling the 0D and 3D models, the hemodynamic numerical simulations of different counterpulsation modes were conducted based on the geometric multi-scale model. We also collected the AP waveforms and CO data of the subjects during the counterpulsation state and compared them with the calculated results of the numerical model. The ratio of the root mean square error (RMSE) between pressure waveforms of the two samples was calculated to verify the validity of the multi-scale model. Since aortic root pressure cannot be non-invasively measured, we collected the pulse pressure of the brachial artery to estimate the AP. This estimating method has been described in our previous study (Li et al., 2020). The brachial artery pressure was collected using a Fukuda pulse wave meter (Song et al., 2016), whereas the CO data were monitored using a color Doppler ultrasound. We performed numerical simulations of the different counterpulsation modes for the three models (standard model, individual 1 and individual 2).

## Results

### The Influence of Pressure Amplitude

**FIGURE 4:**
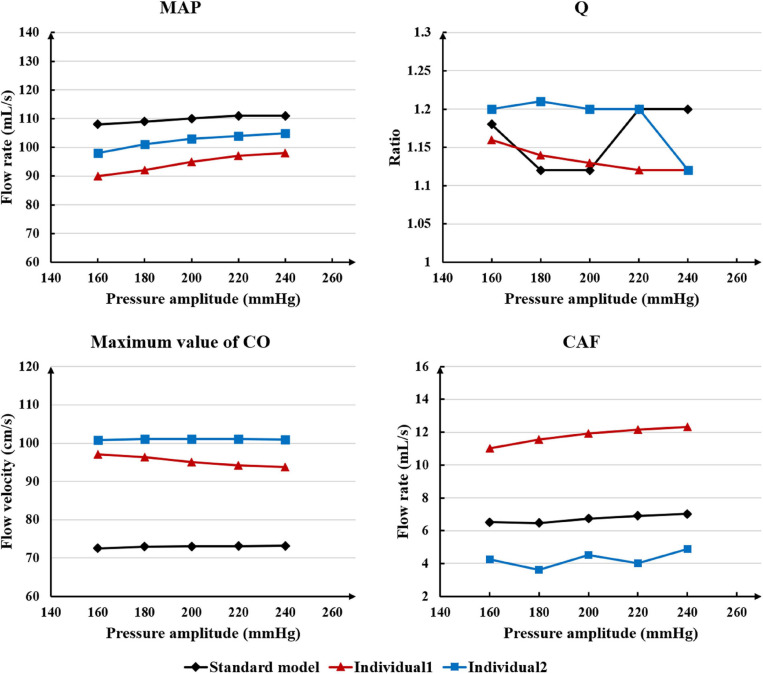
The variations of acute hemodynamic effects under different pressure amplitudes.

**FIGURE 5:**
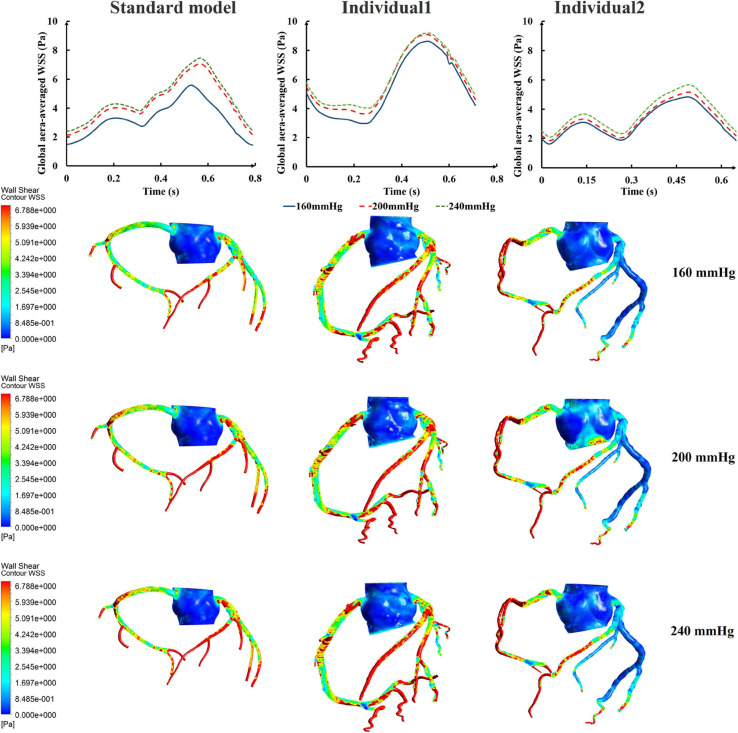
The variations of global area-averaged WSS and the maximum flow moment WSS of three coronary arteries under different pressure amplitudes.

**FIGURE 6:**
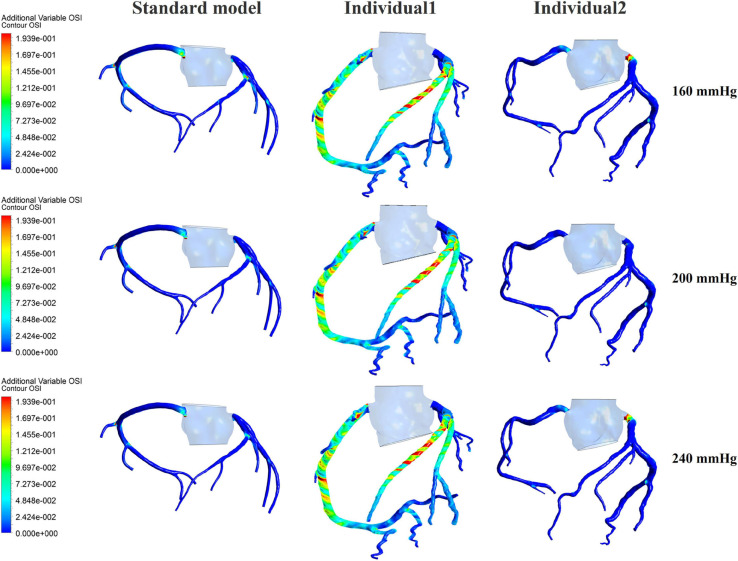
The variations of global OSI of three coronary arteries under different pressure amplitudes.

While maintaining the pressure for a fixed duration (pressure release moment is 0.6 s in a cardiac cycle), the hemodynamic effects of different pressure amplitudes on coronary arteries were simulated. To observe the variations of acute hemodynamic effects, we extracted the mean arterial pressure (MAP), the value of Q (DBP/SBP), coronary artery flow (CAF), and the CO under different counterpulsation modes. Because the blood will flow back to the aortic root during diastole in the counterpulsation state, causing errors in the measurement of the mean value of the CO, the maximum systolic velocity of the CO, which reflects the systolic function of the heart, was extracted for observing the acute effect. The maximum velocity of the CO was calculated by dividing the maximum systolic flow rate by the area of the aortic root. It was found that within the counterpulsation pressure range associated with the clinical applications, the increasing pressure amplitude of the three parts resulted in a slight upward trend in the MAP and the CAF. There was no apparent change in the maximum CO velocity and the value of Q. The results are shown in Figure 4. However, when the pressure was greater than the critical value (approximately 200 mmHg), the acute hemodynamic effects exhibited little improvement. Compared with the resting state, the variations of MAP, Q, maximum CO velocity and CAF of the three models under the pressure amplitude of approximately 200 mmHg are shown in Table 1. As the data show, when the critical pressure amplitude was applied, the increases of MAP and maximum CO velocity were slight compared with the resting state, whereas the increases of Q and CAF were more pronounced. Figure 5 shows the variations in global area-averaged WSS and the maximum flow moment WSS of three coronary arteries (the standard model and the two individual models) with the pressure amplitude. It was observed that when the pressurization duration was fixed, the area-averaged WSS of the global coronary artery slightly increased when the pressure amplitude increased. The influences of different pressure amplitudes on the OSI of the coronary artery inner wall are shown in Figure 6.

**TABLE 1:** Compared with the resting state, the variations of MAP, Q, max CO velocity, and CAF of the three models under the pressure amplitude of approximately 200 mmHg, where IP is increased percentage.

**Model**	**State**	**MAP (mmHg)**	**Q**	**CAF (mL/s)**	**Max CO velocity (cm/s)**
Standard model	Resting	97	0.67	4.40	72.65
	EECP	110	1.12	6.72	73.04
	IP	13.40%	67.16%	52.72%	0.54%
Individual 1	Resting	93	0.66	6.97	84.25
	EECP	95	1.13	11.93	95.04
	IP	2.15%	71.21%	71.16%	12.81%
Individual 2	Resting	103	0.65	3.72	86.76
	EECP	103	1.20	4.51	101.11
	IP	0%	84.62%	21.24%	16.54%

### The Effect of Pressurization Duration

**FIGURE 7:**
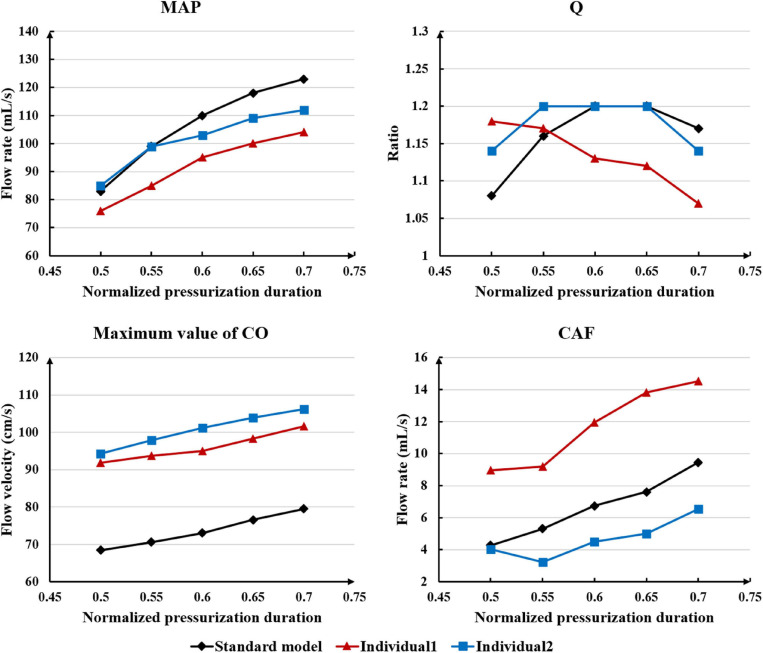
The variations of acute hemodynamic effects under different pressurization durations.

**FIGURE 8:**
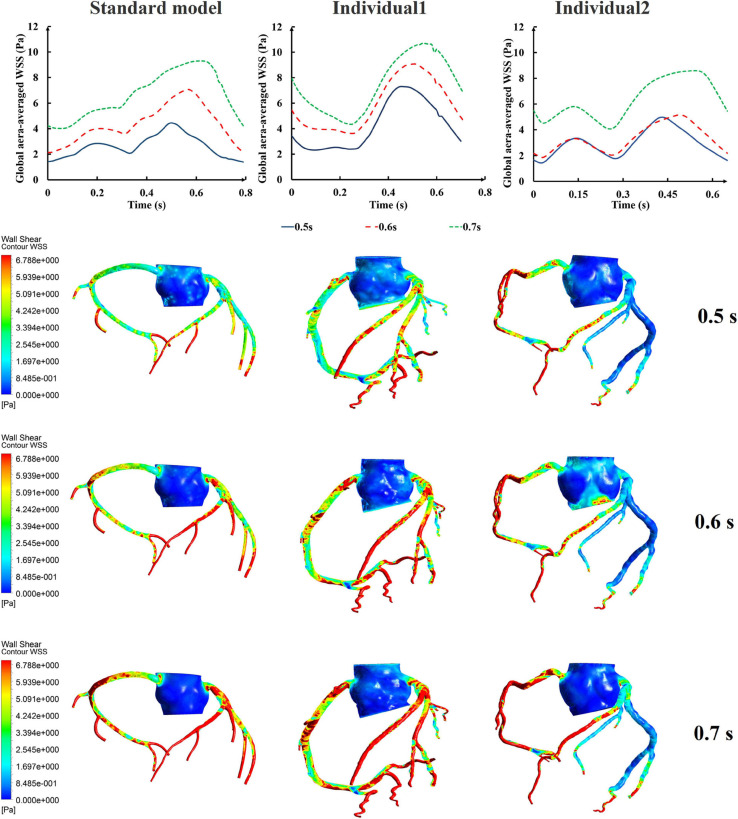
The variations of global area-averaged WSS and the maximum flow moment WSS of three coronary arteries under different pressurization durations.

Differing from the pressure amplitude, the hemodynamic effects of the pressurization duration on the cardiovascular intervention were more obvious. By fixing the starting moment of pressurization (t_*s*_) and changing the pressure release moment (t_*e*_) to simulate different pressurization durations (t_*h*_), it was observed that the MAP, CAF, and maximum CO velocity presented an obvious increase as the pressurization duration increased, whereas the variations of Q did not have a significant law, as shown in Figure 7. When the pressure was released at 0.7 s within a cardiac cycle, the acute variations of MAP, Q, maximum CO velocity and CAF were observed, as shown in Table 2. Compared with the influence of pressure amplitude, the increases of MAP, CAF and maximum CO velocity by longer pressurization duration were greater, whereas the increase of Q was at the same level. Moreover, the global area-averaged WSS for all three coronary artery models presented a more pronounced increase compared with the influence of the pressure amplitude, as shown in Figure 8.

**TABLE 2:** Compared with the resting state, the variations of MAP, Q, max CO velocity, and CAF of the three models when the pressure was released at 0.7 s, where IP is increased percentage.

**Model**	**State**	**MAP (mmHg)**	**Q**	**CAF (mL/s)**	**Max CO velocity (cm/s)**
Standard model	Resting	97	0.67	4.40	72.65
	EECP	123	1.17	9.45	79.50
	IP	26.80%	74.63%	114.77%	9.43%
Individual 1	Resting	93	0.66	6.97	84.25
	EECP	104	1.07	14.53	101.64
	IP	11.83%	62.12%	108.46%	20.64%
Individual 2	Resting	103	0.65	3.72	86.76
	EECP	112	1.14	6.55	106.14
	IP	8.74%	75.38%	76.08%	22.34%

**FIGURE 9:**
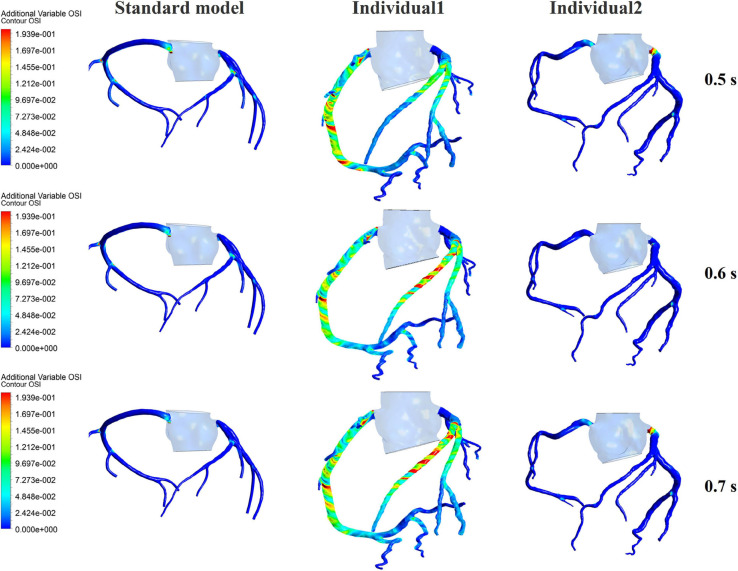
The variations of global OSI of three coronary arteries under different pressurization durations.

The influences of different pressurization durations on the OSI are shown in Figure 9. It can be seen that the increase in both pressure amplitude and pressurization duration had a slight impact on the OSI of the coronary artery. Table 3 shows the average values of time-averaged WSS (TAWSS) and area-averaged OSI of the global coronary arteries under various counterpulsation modes. In addition, to observe the variations of the hemodynamic risk areas of the vascular inner wall (WSS < 1, WSS > 7, and OSI > 0.2), the ratio of the hemodynamic risk areas in the three models under different counterpulsation modes was extracted (see Table 3). We also compared the Q values calculated under the different modes. It can be seen from the results that with increased pressure amplitude, the low WSS and high OSI areas decreased, and the increased pressurization duration significantly increased the excessive WSS area. However, the Q values presented an indistinct law under the different modes.

**TABLE 3:** Variations of TAWSS, OSI, Q value, and the ratio of the hemodynamic risk area in the three models under different counterpulsation modes.

***Model***	***EECP modes***		***TAWSS***	***OSI***	***WSS < 1***	***WSS > 7***	***OSI > 0.2***	***Q***
Standard model	PM	160	3.278	0.00553	5.58%	13.28%	0.55%	1.18
		200	4.364	0.00401	4.87%	17.63%	0.50%	1.12
		240	4.647	0.00368	4.41%	20.34%	0.50%	1.20
	NPRM	0.5	2.618	0.00671	6.57%	11.27%	0.61%	1.08
		0.6	4.364	0.00401	4.87%	17.63%	0.50%	1.21
		0.7	6.612	0.00263	3.53%	44.28%	0.44%	1.17
Individual 1	PM	160	5.388	0.05285	1.64%	16.96%	2.43%	1.16
		200	5.911	0.05307	1.26%	19.25%	2.58%	1.13
		240	6.119	0.05334	1.00%	20.77%	2.67%	1.12
	NPRM	0.5	4.231	0.04714	1.77%	14.31%	2.08%	1.18
		0.6	5.911	0.05307	1.26%	19.25%	2.58%	1.13
		0.7	7.452	0.05247	2.10%	25.97%	3.36%	1.07
Individual 2	PM	160	3.103	0.00614	51.44%	8.41%	0.51%	1.20
		200	3.334	0.00263	48.42%	10.22%	0.13%	1.20
		240	3.681	0.00572	42.94%	12.77%	0.40%	1.12
	NPRM	0.5	2.938	0.00569	55.03%	7.61%	0.38%	1.14
		0.6	3.334	0.00263	48.42%	10.22%	0.13%	1.20
		0.7	6.352	0.00529	28.54%	29.75%	0.36%	1.14

*PM is pressure amplitude and NPRM is normalized pressure release moment (Unit: pressure: mmHg, time: s, WSS: Pa).*

### Clinical Validation Results

**FIGURE 10:**
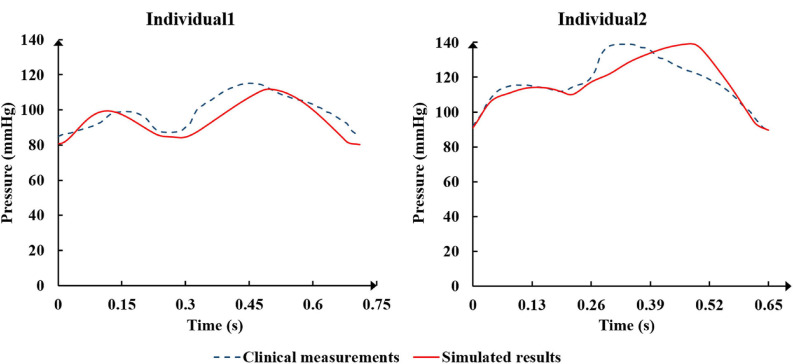
The comparison of aortic pressure waveforms between the clinical measurements and the simulated results from the two individualized model in the resting state and counterpulsation state.

The physiological indicators calculated using the standard and individualized models are shown in Table 4. In the resting state and counterpulsation state, the comparison of aortic pressure waveforms between the clinical measurements and the simulated results using the two individualized models is shown in Figure 10. The ratio of RMSE between pressure waveforms of the two samples were 6.67% and 6.51%. We also compared the waveforms of CO and coronary flow from the standard model with the clinical reports both in the resting state and counterpulsation state, taken from our previous study (Li et al., 2019a, Med. Biol. Eng. Comput.). These results revealed that whether resting or counterpulsation is considered, the physiological indicators calculated by the standard model matched most people’s clinical standards. The indicators calculated by the individual models were consistent with the real measurements obtained from the two subjects. Based on these comparisons, it can be seen that there were some errors between the model-simulated results and the clinical measurements during the counterpulsation state. However, these errors were within an acceptable range.

**TABLE 4:** The clinical physiological data of clinical measurements and simulated results.

**Model**	**State**	**SBP (mmHg)**	**DBP (mmHg)**	**MAP (mmHg)**	**CO/Max CO velocity (L/min or cm/s)**	**Heart rate (n/min)**
Standard model	RCS	120	80	93	5.00	75
	RS	119	76	97	5.65	75
Individual 1	RCM	117	73	94	6.95	82
	RS	115	76	93	6.67	82
Individual 2	RCM	126	81	101	6.33	83
	RS	122	84	102	6.36	83
Individual 1	ECM	99	114	99	98.82	82
	ES	99	112	95	95.04	84
Individual 2	ECM	116	139	118	103.53	90
	ES	114	139	117	106.14	92

*ECM and ES are clinical measurements and simulated results during the EECP state, respectively. RCS, RCM, and RS are clinical standards, clinical measurements, and simulated results in the resting state, respectively.*

## Discussion

### Analysis of Different EECP Modes Affecting Hemodynamics

Based on the results described, the coronary flow and global TAWSS of the coronary arteries slightly increased with increased pressure amplitude. For the standard model, it can be found from Figure 4 that when the pressure was higher than approximately 200 mmHg, minimal change was observed. This result might be due to the effect of the vascular collapse model. The previous study (Li et al., 2019b, Biomed. Eng. Online) had presented the critical external pressure value for the vascular collapse of the external iliac artery in the standard model, which was approximately 200 mmHg. When the pressure was greater than this critical value, the external iliac artery collapsed, and it was difficult to observe any additional effects, even with further increases in pressure amplitude. The critical pressure values for vascular collapse for the three models were not the same due to the different vascular properties and blood pressures of each individual.

Compared with the pressure amplitude, the effect of pressurization duration was more pronounced. The increase in pressurization duration effectively increased MAP, maximum CO velocity and coronary blood perfusion, improved ischemic conditions, increased TAWSS and reduced OSI at the same time. By quantitatively extracting the simulated acute indicators, we considered that these results might have occurred because of the different aortic pressures caused by differing pressurization durations. Table 5 shows the simulated acute indicators, including the mean diastolic blood pressure near the pressurized iliac artery (MDBP-IA), mean diastolic blood pressure (MDBP) of the aorta, SBP and CAF, under three pressurization durations. It was found that when the pressurization duration was insufficient, it could not maintain the prolonged pressure in the blood vessels of the pressurized body. The blood flowing back to the upper body did not have enough time to form a high-pressure state. Consequently, when the cuffs’ pressure was released and a low-pressure load area was formed, the blood in the upper body immediately flowed to the lower body, which resulted in a decrease in the SBP. In contrast, when the pressurization duration was sufficient, the lower body maintained a high-pressure state during diastole. Subsequently, the blood in the upper body was unable to immediately return to the distal areas, which greatly increased the MDBP and increased the maximum coronary blood flow. The coronary arteries (especially the left coronary artery) are primarily supplied during diastole, and the MDBP was increased considerably during the application of EECP. Although the coronary flow autoregulation was considered in this model, as shown in Figure 3, it exceeded the critical perfusion pressure that maintains a constant flow, resulting in the significant increase in coronary flow that was observed.

**TABLE 5:** The simulated acute indicators of the three models under different pressurization durations.

**Model**	**NPRM**	**MDBP-IA (mmHg)**	**MDBP (mmHg)**	**SBP (mmHg)**	**CAF (mL/s)**
Standard model	0.5	80	81	98	4.59
	0.6	111	112	117	6.72
	0.7	127	127	124	9.45
Individual 1	0.5	79	79	81	9.06
	0.6	97	98	100	11.93
	0.7	106	110	111	14.53
Individual 2	0.5	85	85	98	4.04
	0.6	111	107	109	4.51
	0.7	124	116	117	6.55

*NPRM is normalized pressure release moment, MDBP-IA is the mean diastolic blood pressure near the pressurized iliac artery, MDBP is the mean diastolic blood pressure of the aorta, SBP is the systolic blood pressure of aorta, and CAF is the coronary artery flow.*

**FIGURE 11:**
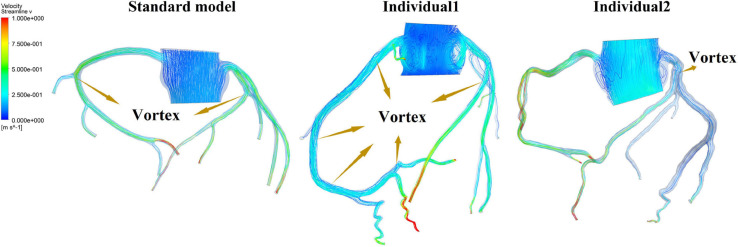
Velocity streamlines of three models in the counterpulsation state.

**FIGURE 12:**
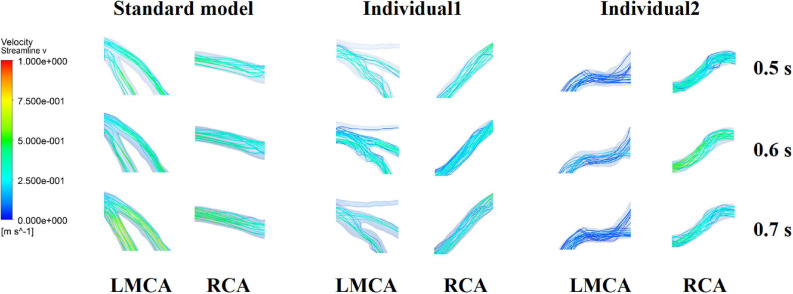
Flow velocity streamlines at the inlet of RCA and LMCA of three models under the different pressurization durations.

It was found from the results in Figures 6, 9, the OSI calculation results of individual 1 were so different from other results. The reasons of the OSI difference include two factors. First, the CO of individual 1 was significantly greater than the others, resulted in more coronary artery flow in the counterpulsation state as shown in Tables 1, 2. Also, the diameter and torsion angle of individual 1 were great than other individuals. More coronary artery flow might result in more possible vortex or irregular flow, especially in the vascular segments with larger curvature and torsion angle like left anterior descending branch (LAD). The velocity streamlines of three models were presented in Figure 11. It was found that in the counterpulsation state, vortex, which is usually the cause of flow oscillation, appeared in several areas of LAD and right coronary artery (RCA) of individual 1. As a result, the OSI results of individual 1 were greater. However, there were only a few areas with high OSI, and the OSI results in most areas were within a reasonable range (<0.2). In addition, the variations of OSI of three models did not present a consistent law as shown in Table 3. We thought that the variation of OSI mainly depended on the flow details at the inlet of coronary artery. Influenced by different EECP modes, more vortex and irregular flow existed at the inlet might result in more flow oscillation in the local areas. The flow velocity streamlines of RCA and left main coronary artery (LMCA) were extracted to explore the reason of OSI variation, as shown in Figure 12. It was found that, vortex of the standard model reduced with the prolonged pressurization duration. For individual 1, significant vortex appeared when pressure released at 0.6 s, and for individual 2, less vortex appeared when pressure released at 0.6 s, which matched the variation of OSI. However, the flow velocity at the inlet depended on the boundary conditions provided by 0D model. With the consideration of coronary autoregulation for each model, the variation of flow boundary of inlet was patient-specific, complex and not regular as the EECP modes changing. As a result, the variations of OSI of three models may not present a regular change.

### Clinical Significance

Judging from the trend in variations in WSS and OSI, it seemed that the increased amplitude and duration of the pressurization were beneficial for the improvement of the hemodynamic environment of the coronary VECs. However, by quantitatively presenting the TAWSS and the ratio of hemodynamic risk areas of the three coronary artery models, it was found that the average TAWSS of the three models was up to 6.612, 7.452 and 6.352 Pa under the maximum pressurization duration, which was around the upper limit of the moderate value (7 Pa). In addition, the average area of excessive TAWSS (>7 Pa) exhibited a high ratio, which accounted for 44.28, 25.97 and 29.75%. This was not a good mechanical state. First, the excessive WSS is more likely to damage the vascular intima and cause pathological expansion of the blood vessel. Furthermore, for patients with coronary heart disease, the WSS in the narrowed region will be significantly higher than the normal area due to the smaller diameter at the stenosis. Especially for patients with vulnerable plaques, excessive WSS can even lead to plaque rupture, resulting in the formation of a thrombus or even acute myocardial infarction and other irreversible consequences (Fu et al., 2015). Under the mode of maximum pressurization duration, the WSS of the stenosis in patients with coronary heart disease was likely to exceed the moderate range, causing adverse effects and risks. Therefore, this study indicated that when EECP is used to treat patients with coronary heart disease, it might not be advisable to adopt a longer pressurization duration during each cardiac cycle. The long-term hemodynamic outcomes of the treatment could be improved by increasing the pressure amplitude.

In addition, the current clinical EECP therapy only uses Q > 1.2 to evaluate the therapeutic effect on patients with coronary heart disease. From the results seen in Table 3, it was observed that the Q value could reach 1.2 under various counterpulsation modes. There was no clear rule for the variations of Q, and the higher Q value might not result in better effects. As mentioned previously, the longer pressurization duration might damage the vascular intima while the Q value was still in an acceptable range. Therefore, this study demonstrated that in clinical treatment, the improvement of the Q value should not be used as the only evaluation index, but other physiological indicators should be considered. Because the pressurization duration significantly affected MAP, and the coronary flow was affected by the CO, it appeared that MAP and CO (or maximum CO velocity) were the correct choices.

### Model Validation

The verification results from the clinical experiments demonstrated that the simulated data of the standard model at the resting state matched the clinical standard physiological indicators well. As a result, the standard model has universal applicability that makes it suitable to study the systemic hemodynamic laws of the blood circulatory system for most people. For each individual, the physiological data (such as AP, CO etc.) need to be collected to individualize the numerical model. The simulated results of the individualized model matched the clinical measurements, indicating the success of the model individualization method. During the counterpulsation state, the blood pressures simulated by the individualized models were closed to the measured pressure waveforms, but there were still some gaps. From the results of area-averaged WSS shown in Figures 5, 8, the time when the maximum WSS of Individual 2 appeared was similar to the maximum pressure moment. Obviously, the simulation error of aortic pressure waveforms indeed affected the peak value time of the calculated results. However, the maximum pressure values and MAP of simulated results matched clinical measurements well, as shown in Table 4. Therefore, the peak value moments of the calculated results had some errors but the mean values were approximately accurate. In addition, the matched heart rate during counterpulsation was the function of the neural regulation model. Therefore, whether considering the model qualitatively or quantitatively, the model established in this study had a potential for effective, accurate and suitable hemodynamic simulation of the human body under resting and counterpulsation states after more patient data were tested.

### Limitations and Future Work

In this study, only two clinical trials were carried out to verify the individualized models. In the future, the universality of the standard model will be verified through multi-center clinical experiments. Current clinical evaluation of the EECP therapeutic effect is based only on the Q value. In future studies, more physiological indicators related to coronary hemodynamic effects will be proposed to develop a better and more comprehensive evaluation method for the treatment effects. Also, the long-term effects of EECP on VECs do not just depend on WSS. The model of cells reaction to WSS (e.g., Ca^2+^ dynamics, eNOS production, etc.) should be considered in future work. Coronary autoregulation is a complex cellular reaction mechanism, which act on a LOCAL basis. Although the autoregulation was considered for coupled 0D model of each coronary branch, the global autoregulation model was adopted in this study, which might result in the insufficient modeling precision. In future work, more actual and complex model that includes the local cellular reaction mechanism will be considered.

## Conclusion

This study used the 0D/3D geometric multi-scale modeling method to investigate the long-term hemodynamic effects of EECP on the vascular intima in coronary artery disease treatment. The model thoroughly considered the neural regulation mechanism of the human body and vascular collapse of the lower body during counterpulsation. It was found that for both acute and long-term effects, the influence of pressurization duration was more significant than pressure amplitude. For patients with coronary heart disease, a longer pressurization duration might result in an excessive WSS, which could easily damage the vascular intima at the stenosis. Therefore, prolonged pressurization duration in a cardiac cycle was not recommended. Long-term treatment outcomes could be improved by increasing the counterpulsation pressure amplitude. Also, more physiological indicators but not a single Q value could be adopted to evaluate the treatment effects. The verification using clinical experiments proved the effectiveness of the individualized numerical model and the value of its application in the numerical simulation of EECP.

## Data Availability Statement

The original contributions presented in the study are included in the article/supplementary material, further inquiries can be directed to the corresponding author.

## Ethics Statement

The studies involving human participants were reviewed and approved by the Medical Ethics Committee of Peking University Third Hospital. The patients/participants provided their written informed consent to participate in this study.

## Author Contributions

BL was responsible for modeling, simulation, data analysis, and manuscript preparation. KX assisted the hemodynamic simulation. JL assisted in 0D modeling. BM assisted in data analysis. NL and HS assisted in the reconstruction of the 3D model. ZZ was responsible for providing experimental data. XZ was responsible for language modification. All authors contributed to the article and approved the submitted version.

## Conflict of Interest

XZ was employed by the Philips (China) Investment Company, Shanghai, China. The remaining authors declare that the research was conducted in the absence of any commercial or financial relationships that could be construed as a potential conflict of interest.
